# Utilising polymorphisms to achieve allele-specific genome editing in zebrafish

**DOI:** 10.1242/bio.020974

**Published:** 2016-11-28

**Authors:** Samuel J. Capon, Gregory J. Baillie, Neil I. Bower, Jason A. da Silva, Scott Paterson, Benjamin M. Hogan, Cas Simons, Kelly A. Smith

**Affiliations:** Division of Genomics of Development and Disease, Institute for Molecular Bioscience, The University of Queensland, Brisbane, Queensland 4072, Australia

**Keywords:** CRISPR/Cas9, Allele-specific, Zebrafish, Genome editing

## Abstract

The advent of genome editing has significantly altered genetic research, including research using the zebrafish model. To better understand the selectivity of the commonly used CRISPR/Cas9 system, we investigated single base pair mismatches in target sites and examined how they affect genome editing in the zebrafish model. Using two different zebrafish strains that have been deep sequenced, CRISPR/Cas9 target sites containing polymorphisms between the two strains were identified. These strains were crossed (creating heterozygotes at polymorphic sites) and CRISPR/Cas9 complexes that perfectly complement one strain injected. Sequencing of targeted sites showed biased, allele-specific editing for the perfectly complementary sequence in the majority of cases (14/19). To test utility, we examined whether phenotypes generated by F0 injection could be internally controlled with such polymorphisms. Targeting of genes *bmp7a* and *chordin* showed reduction in the frequency of phenotypes in injected ‘heterozygotes’ compared with injecting the strain with perfect complementarity. Next, injecting CRISPR/Cas9 complexes targeting two separate sites created deletions, but deletions were biased to selected chromosomes when one CRISPR/Cas9 target contained a polymorphism. Finally, integration of loxP sequences occurred preferentially in alleles with perfect complementarity. These experiments demonstrate that single nucleotide polymorphisms (SNPs) present throughout the genome can be utilised to increase the efficiency of *in cis* genome editing using CRISPR/Cas9 in the zebrafish model.

## Abstract (precis)

**Summary:** Heterozygous single nucleotide polymorphisms in CRISPR/Cas9 target sites bias genome editing in favour of alleles with perfect complementarity to gRNAs, a feature which can be exploited for chromosome-specific editing.

## INTRODUCTION

The clustered regularly interspaced short palindromic repeat (CRISPR)/CRISPR-associated 9 (Cas9) system has recently emerged as the method of choice for genome editing in a wide variety of systems and organisms. The simplicity and versatility of this system, combined with high efficiency and seemingly low off-target effects, have resulted in the rapid uptake of this method. The system consists of the endonuclease, Cas9, guided to prospective target sites by sequence-specific guide RNA molecules (gRNAs), generating double-stranded breaks (DSBs) (Jinek et al., 2012). In addition to applications involving simple locus disruption, successful efforts have been made to co-opt the homology-directed repair (HDR) pathway by including homologous DNA templates whereupon modified or new sequence information can be introduced into the targeted locus (Wang et al., 2013; Yang et al., 2013). Furthermore, a number of studies have demonstrated targeting of variant or disease alleles by CRISPR/Cas9 genome editing, followed by variant repair achieved through HDR (Schwank et al., 2013; Smith et al., 2015; Wu et al., 2013; Yoshimi et al., 2014). Following these initial examples, a flurry of studies have emerged describing mutation correction in both induced pluripotent stem cells (iPSCs) and *in vivo* models of disease. Given the broad range and potential therapeutic applications of this technology, considerable effort has been made to expand the capabilities and applications of CRISPR/Cas9 during genome editing (Fu et al., 2013, 2014; Hsu et al., 2013; Moreno-Mateos et al., 2015; Ran et al., 2013; Slaymaker et al., 2016). One important aspect of CRISPR/Cas9 targeting is that base pair mismatches between gRNAs and their target sequences significantly alter editing activity (Deveau et al., 2008; Jinek et al., 2012; Sapranauskas et al., 2011).

In the zebrafish, CRISPR-based technologies have been used for both generating indels and, with greater difficulty, to induce HDR and introduce exogenous sequences. The low efficiency of HDR, in particular, represents a challenge for the zebrafish research community, making applications such as generation of conditional alleles or specific selective amino acid changes, slow and costly. Several recent reports have investigated the specificity of the CRISPR/Cas9 system in targeting polymorphic sites in diploid genomes (e.g. to revert specific mutations) and find that CRISPR/Cas9 efficacy can be modulated at polymorphic sites within the genome (Smith et al., 2015; Wu et al., 2013; Yoshimi et al., 2014). To take advantage of the site specificity of CRISPR/Cas9 targeting to build more capabilities in zebrafish genome editing, we have used two whole genome sequenced zebrafish strains, for which we have established strain-specific reference genomes, and tested the utility of polymorphism directed targeting. Using these lines, we conducted a genome-wide survey to identify putative CRISPR/Cas9 target sites that contain strain-specific single nucleotide polymorphisms (SNPs). For 19 of these loci, we have experimentally determined their effect on genome editing efficiency. We find that SNPs within the target site insulate genome editing, biasing editing in favour of perfect complementarity between gRNA and target site in the majority of examples. This approach can be applied to internally control F0 induced CRISPR/Cas9 phenotypes, to integrate loxP sites at *in cis* positions on a chromosome and to generate large genomic deletions between *in cis* polymorphic sites. Hence, polymorphism-directed genome editing can be used to improve the utility of the zebrafish model for genetic studies.

## RESULTS AND DISCUSSION

### Identification of strain specific CRISPR/Cas9 sites in two distinct zebrafish strains

It has previously been demonstrated that single base pair substitutions in a CRISPR gRNA, resulting in imperfect complementarity between the gRNA and target site, can reduce or even abolish the efficacy of CRISPR/Cas9-mediated editing (Deveau et al., 2008; Fu et al., 2013, 2014; Hsu et al., 2013; Jinek et al., 2012; Sapranauskas et al., 2011). We reasoned that this feature could be exploited to specifically target sites in the genome that were polymorphic between strains, resulting in the targeting of one strain whilst insulating against genome modification in the other. To identify such polymorphic sites, we utilised the sequenced genomes from two different strains of zebrafish established in-house for a forward genetic screen (Koltowska et al., 2015). The qWIK strain is descendent from a single mating pair of WIK strain adults (Rauch et al., 1997) that were sequenced to over 30-fold coverage across the genome, accounting for the majority of genetic variation in the resulting line. The transgenic *Tg(lyve1:DsRed2)^nz101^* line used in our screens (Okuda et al., 2012) was generated in the AB background (originating from the Zebrafish International Resource Center, Oregon) (Johnson and Zon, 1998; J. Astin, The University of Auckland, Auckland, New Zealand, personal communication; herein referred to as the AB strain) and derived from a genetic bottleneck of approximately five founder fish. These lines have been subsequently maintained by in-crossing multiple pairs of descendants to retain a genetically homogeneous gene pool for each strain. The pooled DNA from six male descendants of the founder fish were simultaneously sequenced to achieve over 30-fold coverage across the genome, giving a representative account of the genomic variation within this strain. Although bottlenecked, these lines are not isogenic (which results in a well-recognised breeding depression in zebrafish) and are considered representative of the WIK and AB strains.

The *Streptococcus pyogenes* type II CRISPR-Cas complex targets sites with a protospacer adjacent motif (PAM), defined as 5′-NGG, and therefore any GG dinucleotide occurring on either strand of the genome represents a potential CRISPR/Cas9 target site. Analysis of the Zv9 genome identified 94,188,783 potential CRISPR/Cas9 target sites. Using the strain-specific reference genome sequences of the qWIK and AB strains, we identified 3,825,151 ‘strain-specific’ loci, where a strain-specific polymorphism is located in either the PAM sequence or putative gRNA sequence. We hypothesised these strain-specific CRISPR/Cas9 target sites could be used to target or enrich editing to a chosen strain-specific allele.

We found that 37,259 unique coding exons from 12,351 unique genes contain one or more ‘strain-specific’ loci, meaning that the coding regions of over 46% of annotated genes are potentially targetable in a strain-specific manner using our pair of zebrafish strains. If intronic regions are also considered, we found that 17,581 (66%) unique genes contain at least one ‘strain-specific’ CRISPR/Cas9 target site. Furthermore 57% of genes contain ‘strain-specific’ CRISPR/Cas9 target sites that flank one or more coding exons.

### Allele-specific genome editing

**Fig. 1.**
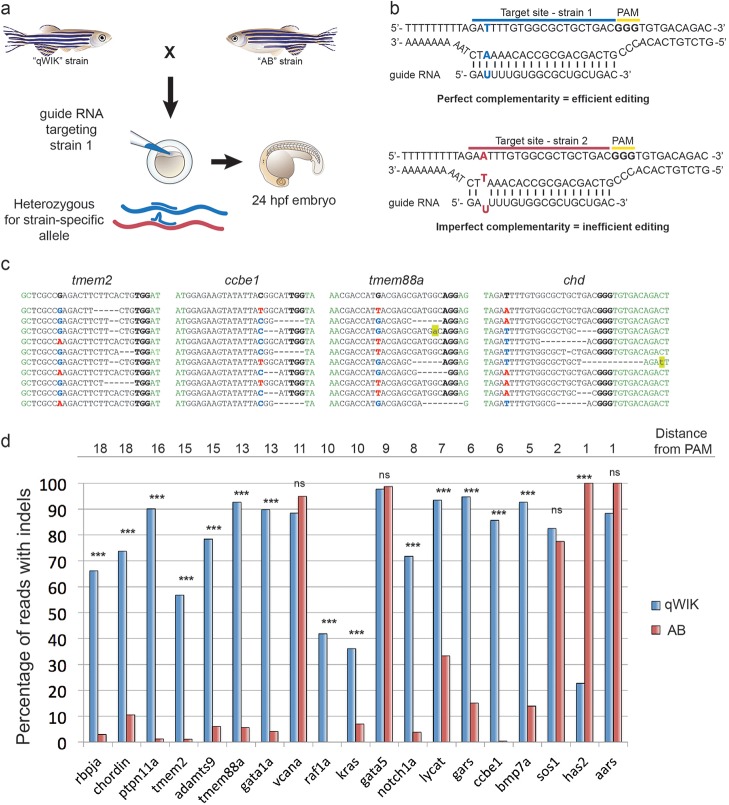
**Heterozygous polymorphisms within CRISPR/Cas9 target sites bias genomic editing towards alleles with perfect complementarity to the gRNA.** (A) Experimental scheme testing editing efficiency of various gRNAs. All gRNAs were targeted to polymorphic target sites, where the gRNA had perfect complementarity to the qWIK allele, resulting in mismatches with the AB allele. Red, AB strain; blue, qWIK strain. (B) An example of a polymorphic target site (within the *chordin* locus) demonstrating perfect complementarity between the gRNA and the qWIK strain (blue) versus imperfect complementarity in the AB strain (red). (C) Examples of editing from deep sequencing of PCR products amplified across a number of target sites (*tmem2*, *ccbe1*, *tmem88a* and *chd*) showing positive editing bias for the qWIK allele. Top row is the reference sequence, with the qWIK variant depicted and the PAM in bold. Polymorphic sites are depicted as bold blue for the qWIK allele and bold red for the AB allele. Deletions are represented by dashes, and insertions/mutations are highlighted in yellow. (D) Bar graph depicting the percentage of edited over total reads for each allele. The order of target sites is arranged from SNPs that are furthest from the PAM to closest to the PAM, as indicated above the graph. *P*<0.001, as determined by Chi squared test; ns, not significant.

To test whether these strain-specific polymorphisms could be used for allele-specific genome editing, we created 19 different gRNAs targeting sites specific to the qWIK genome and injected them into embryos derived from a cross between the qWIK and AB strains. Injected embryos were collected at 1 day post fertilization (dpf), DNA extracted and the targeted region amplified by PCR and sequenced. Out of the 19 gRNAs, 14 created indels in the qWIK allele with higher efficiency than the AB allele (Fig. 1). Of the remaining five gRNAs, four showed equally high efficiency for both alleles and only one showed a bias for cutting the AB allele. Our results are consistent with previous reports describing differing rates of efficiency and specificity between gRNAs (Fu et al., 2013, 2014; Gagnon et al., 2014; Hsu et al., 2013; Moreno-Mateos et al., 2015), however the majority of gRNAs exhibited allelic bias providing utility in the genetic applications discussed below.

### Controlled phenotypic screening using CRISPR/Cas9 editing

**Fig. 2.**
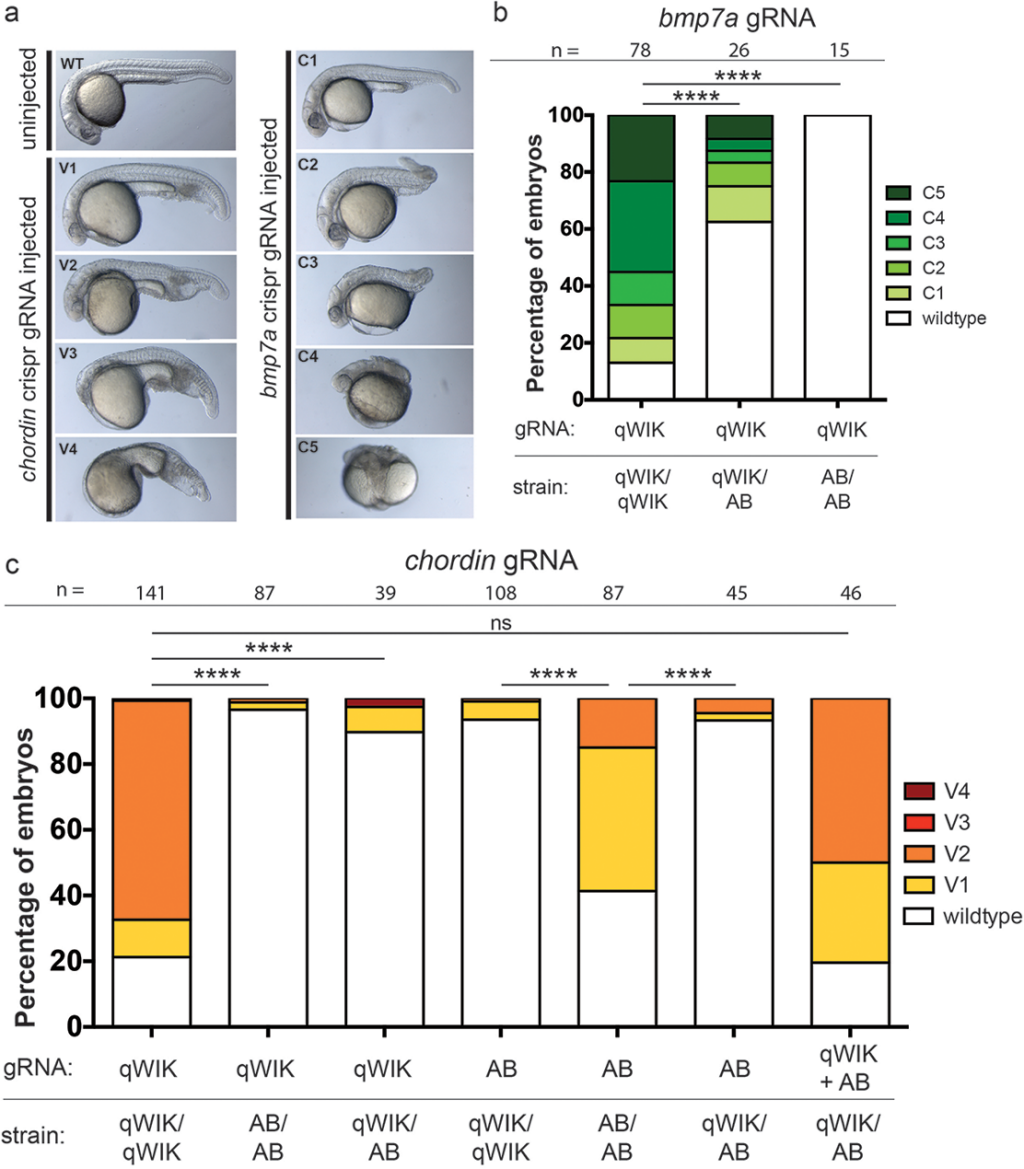
**Heterozygous polymorphisms within CRISPR/Cas9 target sites can internally control recessive phenotypes generated in F0 injected embryos.** (A) Bright field lateral view images of 1 dpf embryos uninjected or injected with gRNAs and *cas9* mRNA to target the *chordin* or *bmp7a* locus. *chordin* gRNA injected embryos manifested a range of ventralised phenotypes (V1-V4), and *bmp7a* gRNA injected embryos a range of dorsalised phenotypes (C1-C5) (Mullins et al., 1996), consistent with biallelic disruption of these dorsoventral patterning genes. (B) Graphical representation of the phenotypic categories of embryos injected with a ‘qWIK-specific’ gRNA into either a qWIK incross (qWIK/qWIK), a qWIK crossed with AB (qWIK/AB) or AB incross (AB/AB). Imperfect complementarity reduced the frequency of dorsalised phenotypes. (C) Graphical representation of ventralised phenotypes following *chordin* gRNA/Cas9 mRNA injection into different strain crosses. ‘qWIK-specific’ into qWIK/qWIK, ‘AB-specific’ into AB/AB or co-injection of ‘qWIK-specific’ and ‘AB-specific’ gRNAs into qWIK/AB cross resulted in a high proportion or embryos with ventralised phenotypes whereas very few embryos with ventralised phenotypes were observed for injection of ‘qWIK-specific’ into AB/AB or qWIK/AB or reciprocally ‘AB-specific’ into qWIK/qWIK or qWIK/AB. Total number of embryos injected for each category is represented above graphs. In B and C, ****P<0.0001, as determined by Fishers exact test; ns, not significant.

Several reports have emerged describing the utility of biallelic, CRISPR/Cas9-mediated genome editing as a method for phenotypic screening (Shah et al., 2015; Varshney et al., 2015). With the potential for off-target effects or confounding RNA-induced toxicity, it could be argued that this approach is no more specific than morpholino-based screening. Given this shortcoming, we hypothesised that the allelic bias observed by the majority of gRNAs tested could be exploited as an internal specificity control for CRISPR/Cas9-based phenotypic screening. To test this theory, we generated gRNAs against the dorsoventral patterning genes, *bmp7a* and *chordin*, and used their respective dorsalised and ventralised loss-of-function phenotypes as read-outs of CRISPR/Cas9 efficiency. We designed our gRNAs to target sites harbouring a polymorphism between the qWIK and AB strains. In the first instance, we injected the ‘qWIK-specific’ *bmp7a* gRNA/Cas9 mRNA cocktail into clutches from qWIK incrosses, AB incrosses or qWIK crossed to AB and scored for phenotypes at 1 dpf. Consistent with previously published mutant phenotypes (Dick et al., 2000; Schmid et al., 2000), a range of dorsalised phenotypes were observed in injected embryos when using the gRNA with perfect complementarity to the target site, suggesting biallelic cutting for this recessively acting gene (Fig. 2A,B). However, upon injection of the gRNA into the reciprocal strain (i.e. ‘qWIK-specific’ into AB strain) or into a clutch from qWIK crossed to AB, these phenotypes were reduced in number (Fig. 2B). We next tested the *chordin* locus but this time used a ‘qWIK-specific’, an ‘AB-specific’ gRNA or co-injection of the two gRNAs. As was observed for the bmp7a targeting, we saw phenotypes resembling the published ventralised *chordin* mutant phenotypes when injecting gRNAs with perfect complementarity to the target sequence (Schulte-Merker et al., 1997). This included either ‘qWIK-specific’ gRNA into qWIK incross, ‘AB-specific’ gRNA into AB incross or co-injection of qWIK and AB gRNAs into qWIK crossed to AB strain. For all other combinations, ventralised phenotypes were significantly reduced. These results demonstrate that, when present heterozygously, a SNP in the target site is sufficient to internally control for phenotypes of recessively acting genes generated by CRISPR/Cas9 injection into F0 embryos. This approach could be used for blind, genotyping-based validation of transient phenotypic screens using CRISPR/Cas9. In addition, the reduced phenotypic severity upon injection of heterozygotes is likely to improve the survival of genome-edited individuals, ultimately assisting in germline transmission for the creation of stable genetic mutants.

### Chromosome-specific genomic deletions

**Fig. 3.**
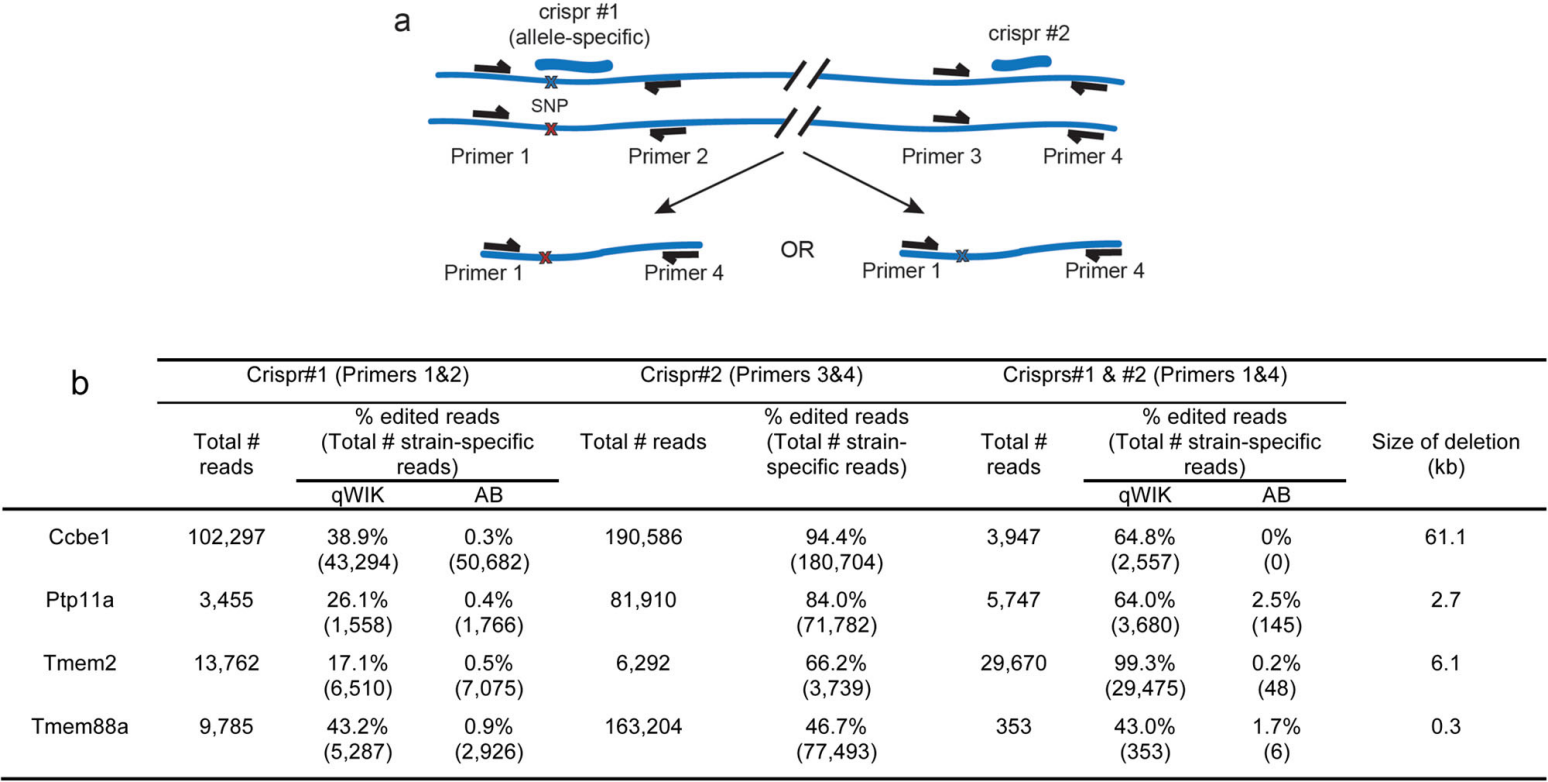
**Chromosome-specific deletions created using CRISPR/Cas9 allelic bias.** (A) Schematic representation of experiment generating genomic deletions using two CRISPR/Cas9 gRNAs, where ‘crispr#1’ targets a polymorphic site and ‘crispr#2’ targets a non-polymorphic site. Blue crosses indicate qWIK-specific SNP. Red crosses indicate AB-specific SNP. (B) Table depicting the editing efficiency for the various gRNAs injected in isolation or in combination and quantified by sequenced PCR products spanning each edited site alone or the deleted regions. Total reads represents all mapped reads (qWIK-specific, AB-specific and unequivocal). Percentage of edited reads for crispr#1 only (primers 1&2) and #2 only (primers 3&4) expressed as a function of total number of strain-specific reads. Percentage of edited reads for crisprs#1 and #2 (primers 1&4) is calculated as a function of total read number.

Another application of CRISPR-mediated double-stranded breaks is to target two genetically linked loci simultaneously to excise a bulk of DNA, creating large genomic deletions (Varshney et al., 2015). This approach can be applied to remove the coding sequence of a gene of interest, or non-coding regulatory elements for the study of gene regulation. To determine whether we could utilise the targeting bias to create deletions on a chosen chromosome, we selected four gRNAs that were efficient and discriminating for the qWIK strain and co-injected these with a second gRNA targeting a distant non-polymorphic site (distance ranging from 290 to 61,163 bp away). We injected one or both gRNAs into embryos from a qWIK/AB cross and PCR amplified across a single target site (for the single gRNA injection) or amplified for fragments that could only be generated if a deletion had occurred (injection of two gRNAs). From sequencing the products, we found that in all four cases deletions were generated almost exclusively in the qWIK strain (>97% reads) (Fig. 3). While this approach does not test the efficacy of the method in terms of germ-line transmission rates, it demonstrates that allele-specificity of gRNAs can be utilised to create deletions on specific chromosomes of interest.

### Chromosome-specific insertion of loxP sites

**Fig. 4.**
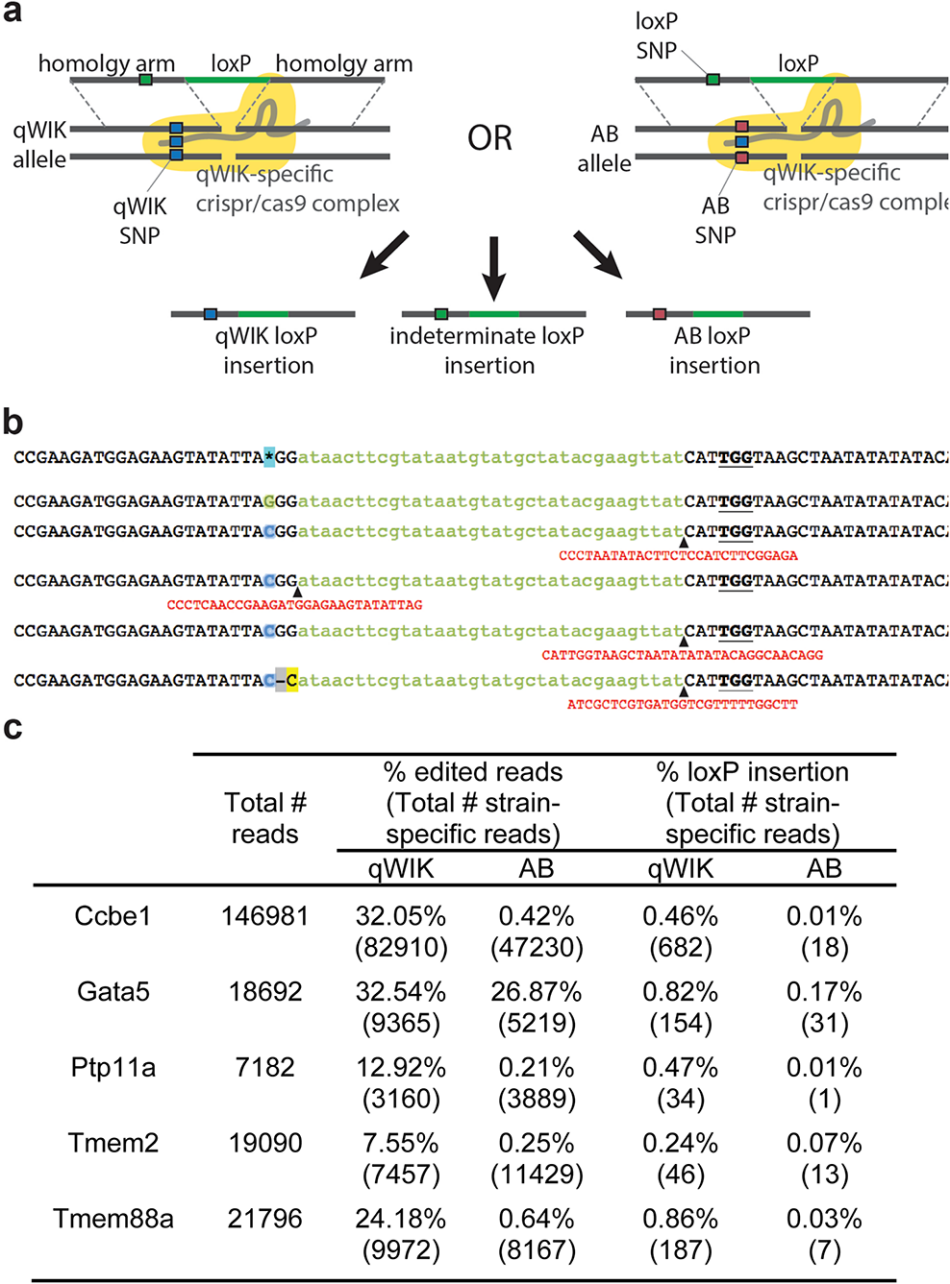
**Allelic-specific insertion of loxP sites.** (A) Schematic of loxP insertion possibilities. CRISPR/Cas9 gRNAs targeting the qWIK allele were injected into qWIK/AB incrosses, along with loxP sites harbouring 33 nucleotide homology arms. Homology arms spanned the strain-specific SNP, therefore a loxP-specific SNP was included to distinguish (and disregard) recombination events that are inconclusive. Green boxes represent loxP-specific SNP; blue boxes, represent qWIK-specific SNP; pink boxes represent AB-specific SNP; green bars represent loxP sequence; yellow shading represents Cas9 enzyme. (B) Example reads from the *ccbe1* locus, demonstrating loxP site insertions that show the loxP SNP (G – green font) or the qWIK SNP (C – blue font). LoxP sites indicated in lowercase green, additional insertions indicated in red (with insertion points indicated with black arrowhead), PAM bold underline, deletion dashed and grey and substitution mutation coloured yellow. (C) Table summarizing results from loxP insertion experiments. Total reads represents all mapped reads (qWIK-specific, AB-specific and indeterminate). Percentage of edited reads as a function of total number of strain-specific reads. Percentage of loxP efficiency for specific strain is calculated as a function of total read number.

As well as creating small and large deletions by non-homologous end joining (NHEJ), genome editing has been used to enhance the integration of specific sequences via the promotion of HDR at targeted sites in the genome. One significant weakness of zebrafish genetics is a lack of conditional and tissue-specific knockout models. An approach that would benefit greatly from chromosomally targeted integration is the insertion of loxP sites *in cis* at selected locations in genes of interest. Using conventional CRISPR/Cas9-assisted integration methods, insertion is likely to occur with a 50% chance of achieving integration *in cis* versus *in trans* and with low rates of integration, achieving two sites *in cis* may prove challenging. To test if allele-specificity could be used to target a specific chromosome for loxP insertion, we utilized qWIK-specific gRNAs that had proven discrimination in their targeting efficiency, as well as one that had been promiscuous at editing both alleles (*gata5* gRNA). These gRNAs were co-injected with single-stranded oligonucleotides, consisting of loxP sites and 33 bp homology arms, to each CRISPR/Cas9 target site. Within the designed homology arms, we also incorporated a SNP specific to the loxP oligo to discriminate in the analysis whether any resultant loxP insertion was attributable to the qWIK, AB or indeterminate allele. Following injection of CRISPR/Cas9/oligo cocktails, embryos were grown for 1 day, DNA extracted, PCR performed across the target site and products deep sequenced. Sequencing data demonstrated that loxP integration was achieved in all five cases, with efficiencies ranging from 1.4% up to 22.3%, in keeping with reports from other laboratories (Auer and Del Bene, 2014). The majority of reads possessed either the ‘loxP SNP’ or harboured a deletion at the diagnostic SNP, and therefore were unable to be categorised as qWIK or AB; however, for those that could be determined, all five cases (including the *gata5* site) showed bias for loxP site integration towards alleles that perfectly complemented the gRNA (Fig. 4). This data demonstrates the capability of allele-specific targeting to bias the insertion of loxP sites into a particular chromosome. To employ this method to maximum efficiency, one would use a homozygous strain (such as qWIK) and target a non-discriminating CRISPR/cas9 site to introduce a loxP site. Once this has been integrated, the homozygous fish can then be outcrossed to an alternate strain (such as AB) and the heterozygous progeny targeted against a discriminating, qWIK-specific CRISPR/Cas9 site, achieving *in cis* integration. Given the current rates of integration, this approach may effectively double the efficiency of generating *in cis* integration of sequences of interest into the genome.

To summarise, we have described two strains of zebrafish that are genomically distinct from the reference zebrafish strain insofar as they provide strain-specific SNPs that can be utilised for CRISPR/Cas9-mediated genome editing in an allele-specific manner. By exploiting the editing bias at these loci, SNPs can be used to internally control for transient phenotypic screening of phenotypes, to create chromosome specific deletions and to enhance the efficiency of loxP insertion *in cis*.

## MATERIALS AND METHODS

### Zebrafish husbandry and experimentation

Animal work followed the guidelines of the animal ethics committee at the University of Queensland, Australia. Zebrafish (*Danio rerio*) were maintained, collected, and staged as described (Kimmel et al., 1995). All embryos were raised at 28.5°C. Injections into phenotypically wild-type embryos were performed using Queensland WIK (qWIK) (Koltowska et al., 2015) and *Tg(lyve1:DsRed2)^nz101^* (Okuda et al., 2012) strains. The qWIK strain was at its fourth generation of inbreeding by the time of publication and therefore already suffering from inbreeding depression. Whilst the authors are willing to disseminate the strain upon request, the simplicity and expense of producing a similarly sequenced (30 times coverage) inbred line would approximate that of shipment fees. Given that a newly generated line would have the added advantage of being in its first generation, we recommend this approach.

### Strain-specific CRISPR/Cas9 site identification

Strain-specific reference genomes were generated as described in (Koltowska et al., 2015). CRISPR/Cas9 target sites were defined as any sequence meeting the criteria N_20_-NGG and were identified in each strain using a custom application crisprfindercocoa (available at https://bitbucket.org/gregonomic/crisprfindercocoa). CRISPR/Cas9 sites were filtered, scored using criteria described in (Montague et al., 2014) and counted using custom Python scripts (found in the utilities directory at the above repository) and the BEDTools toolkit (Quinlan and Hall, 2010). Sequences of the 19 strain-specific CRISPR/Cas9 target sites are detailed in Table S1 and sequences for the non-polymorphic second target site used in the deletion experiments, see Table S2.

### gRNA design and synthesis

gRNA templates were generated as described (Gagnon et al., 2014) with minor alterations to the described protocol. All oligos were obtained from Integrated DNA Technologies (Coralville, IA, USA) as standard primers. Where appropriate, the first two bases of the gRNA were altered to a GG or GA dinucleotide for transcription with T7 or SP6 poylmerase, respectively. gRNAs were synthesised using MEGAScript T7 or SP6 transcription kits (Ambion), purified using the ‘RNA clean & concentrator’ kit (Zymo Research, Irvine, CA, USA) and stored at −80°C.

### Microinjection

Injection mixes were prepared by combining 1 μg of zebrafish codon optimised Cas9 mRNA (Jao et al., 2013) with 500 ng of each gRNA and 0.5 μl Phenol Red (Sigma-Aldrich) in 2.5 μl total volume. 1 nl of each injection mix was injected into the yolk of 1–2-cell-stage embryos. For loxP HDR injections, 100 μM of each loxP oligo (sequence detailed in Table S3) was diluted 1:10 in phenol red and 0.5 μl of this used in place of phenol red in the above injection mix. 1 nl of these injection mixes were injected into the cell of 1-cell-stage embryos.

### DNA extraction

Embryos were grown up at 28.5°C and collected in methanol at 1 dpf. Genomic DNA was extracted from 10 individual embryos and subsequently pooled. This was repeated on a separate injection day to generate replicate samples. DNA extraction was performed by incubation in 50 μl DNA extraction buffer (50 mM KCl, 2.5 mM MgCl_2_, 10 mM Tris pH 8.3, 0.45% IGEPAL, 0.45% Tween20, 0.01% gelatine and 100 μg/ml proteinase K) at 55°C for 60 min followed by 99°C for 15 min. For each sample, at least two different injection sessions were performed and the two biological replicates kept separate. DNA was kept at −20°C for long-term storage.

### Amplicon library preparation and sequencing

A two-step PCR approach was used to prepare sequencing libraries. Primary sequencing primers with sequencing adaptors were designed to amplify 250-450 bp surrounding each target site. Primary PCR products were purified using a 0.9× ratio of magnetic beads (AxyPrep Mag PCR clean up kit). Non-overlapping primary PCR products were combined and diluted to 1 ng/μl to prepare primary PCR libraries. A secondary PCR using Nextera XT indexing primers (Illumina, Inc., San Diego, CA, USA) was carried out using 1 μl of each primary library. Secondary PCR products were purified using a 0.8× ratio of magnetic beads (AxyPrep Mag PCR clean up kit) to produce final sequencing libraries. All PCRs were performed using Phusion high-fidelity DNA polymerase (New England BioLabs, MA, USA). Libraries were sequenced on a MiSeq instrument (Illumina), using 2×300 bp v3 chemistry. Replicate libraries were sequenced on different runs.

### Read trimming, merging, and alignment

Reads were trimmed using Trimmomatic (Bolger et al., 2014) in paired-end mode, using the settings: ILLUMINACLIP: TruSeq3-SE.fa:2:30:10 LEADING:5 TRAILING:5 SLIDINGWINDOW:10:30 MINLEN:75. Paired trimmed reads were merged using usearch (Edgar, 2010), with settings: -fastq_truncqual 3 -fastq_maxdiffs 0 -fastq_minovlen 50. Merged reads were mapped against the *Danio rerio* genome (UCSC assembly version danRer7/Zv9, http://genome.ucsc.edu/cgi-bin/hgGateway?db=danRer7) using BWA-mem (Li and Durbin, 2010) v0.7.5a, and alignments were processed using samtools (Li et al., 2009) v0.1.19 and Picard (http://broadinstitute.github.io/picard/) v1.120.

### Variant counting and statistics

Variant counting was performed using a custom Python script (crispr_amplicon_counter.py, https://bitbucket.org/gregonomic/ampliconseq/). In short, contigs overlapping the CRISPR-targeted region were counted, assigned to the qWIK- or AB-specific allele where appropriate, and assessed for whether they contained insertions and/or deletions covering the targeted region (and if so, their number and length). For loxP insertions, reads were counted as containing the loxP sequence if they contained 10-mer of the loxP sequence (5′-ATAACTTCGTATAGCATACATTATACGAAGTTAT-3′). Statistical analysis was performed using Chi Squared test (Fig. 1) and Fishers exact test (Fig. 2) by comparing wild type versus embryos displaying a phenotype, using Prism 7 software (GraphPad).

### Embryo imaging

1 dpf embryos were mounted laterally in 3% methyl cellulose and imaged on a M165 FC stereo microscope with a DFC425 C camera (Leica).
